# Room-level occupant counts and environmental quality from heterogeneous sensing modalities in a smart building

**DOI:** 10.1038/s41597-019-0274-4

**Published:** 2019-11-26

**Authors:** Jens Hjort Schwee, Aslak Johansen, Bo Nørregaard Jørgensen, Mikkel Baun Kjærgaard, Claudio Giovanni Mattera, Fisayo Caleb Sangogboye, Christian Veje

**Affiliations:** University of Southern Denmark, Campusvej 55, 5230 Odense, Denmark

**Keywords:** Energy modelling, Energy efficiency, Energy infrastructure, Civil engineering

## Abstract

The research areas of occupant sensing and occupant behavior modeling are lacking comprehensive public datasets for providing baseline results and fostering data-driven approaches. This data descriptor covers a dataset collected via sensors on room-level occupant counts together with related data on indoor environmental quality. The dataset comprises 44 full days, collated in the period March 2018 to April 2019, and was collected in a public building in Northern Europe. Sensor readings cover three rooms, including one lecture room and two study zones. The data release contains two versions of the dataset, one which has the raw readings and one which has been upsampled to a one-minute resolution. The dataset can be used for developing and evaluating data-driven applications, occupant sensing, and building analytics. This dataset can be an impetus for the researchers and designers to conduct experiments and pilot studies, hence used for benchmarking.

## Abstract (summary)

**Table:** 

Measurement(s)	carbon dioxide • humidity • visible light energy • occupant count • temperature of air • indoor airflow
Technology Type(s)	sensor • digital camera • damper position
Sample Characteristic - Organism	Homo sapiens
Sample Characteristic - Environment	office building
Sample Characteristic - Location	Kingdom of Denmark

Machine-accessible metadata file describing the reported data: 10.6084/m9.figshare.9971549

## Background & Summary

Accurate estimates of occupant counts in a building can be used in various applications areas, including smart spaces, safety and evacuation, facility management, and building operations^1,2^. In the building operation area, occupant counts can enable applications, like, adaptive ventilation in rooms, occupant-based energy benchmarking, and model-predictive control of room setpoints. In these applications, the more accurately the numbers of occupants can be sensed, the more energy-efficient a building can be operated^3^. For studies of adaptive ventilation and model-predictive control, it is also necessary that occupant presence can be linked to indoor environmental quality and ventilation rates.

Datasets are needed for such research which captures the conditions in non-laboratory settings. This is because the developed systems and algorithms have to handle omissions and faults in the sensor data that they process to be applicable beyond the lab. Therefore, the released dataset is collected in a living lab building, which is a standard building in normal use but where additional effort has been made to enable data collection. However, as standard components are used uncertainties around systems and their calibration is higher than in a laboratory setting.

The building considered for this data release is a teaching and office building, located at the University of Southern Denmark, Odense campus. Denmark has a temperate climate representative for the northern part of Europe. The building has been designed to serve as a living lab for data-driven research on building operation and optimization^4^. The role as a living lab has been communicated at the university, and a screen at one of the entrances show examples of data collected. However, as the data collection is mostly invisible to occupants, the collection does not impact their behavior in any noticeable manner. The building is approximately 8500 m^2^ split across three floors and a basement. It has 1000 occupants on a typical weekday and is used for both student activities and staff. Data is collected at the spatial granularity of rooms. The data have been collected in three rooms, two of them study zones, and one is a lecture room. The rooms were selected to cover different usage patterns and differences in sunlight by facing either south-east or north-west. The study zones have a mixed-use for student activities, such as project work and solving exercises. The teaching room is mostly used for scheduled activities, typically spanning between two to four hours.

The rooms contain a unique collection of sensor modalities covering both occupant presence and indoor environmental quality factors, including CO_2_ concentration level, relative humidity, illuminance, occupant counts, occupant counts entering and leaving the rooms, temperature, and the in-room airflow, estimated by the damper position, which is correlated to the airflow, the air is outdoor air heated using heat recovery. The placement of the sensors follows the standard practice of the building industry in Denmark.

Compared to existing sensor-based datasets for buildings, most of them consider residential homes (e.g., Barker *et al*.^5^). We, however, With this dataset, consider commercial buildings. Previous datasets for commercial buildings include fewer sensor modalities and has a lower temporal resolution than the presented dataset. Previous datasets include a dataset with only three modalities and a small temporal range by the University of Southern Denmark, described in^6^, a dataset by Lawrence Berkeley National Lab with lower granularity and fewer modalities but with more background variables^7^ and a dataset with only one sensor modality by University of Texas, San Antonio^8^. Thereby, the released dataset is unique due to the number of sensor modalities available.

The sensor modalities in the dataset enable researchers to both study new technical solutions (e.g., CO_2_-based occupant estimation algorithms^9^, adaptive ventilation, or model-predictive control) and establish knowledge on occupants and indoor environmental quality (e.g., quantify the correlation between occupants and air quality). The dataset can also be used to learn modeling parameters for occupants to more accurately parameterize building performance simulations^3^.

The occupant counts entering and leaving the rooms have been collected using six state-of-the-art PC2 3D stereo vision cameras produced by the company Xovis, which have been mounted over the entrances to the rooms. To estimate the number of occupants in the rooms, we have used the PLCount algorithm^10^ on the raw readings from the cameras. The sensor and method have been evaluated in the building with a manually obtained ground truth based on video recordings. The study documented in^10^ showed an accuracy of 0.075 Root Mean Square Error (RMSE). The other building data is collected via standard-grade sensors connected to a building management system (BMS), which for the particular building is a Schneider Electric BMS. The data for the release is collected through application programming interfaces of the BMS. The CO_2_ sensor data have not been cleaned. Therefore users of the data should address known issues with this stream, including offsets and drifts^9^. An overview of the different sensor streams can be found in Table 1, including units and uncertainties. See Table 2 for details of the sampling strategies of the various sensors and room. The physical placement of the sensors can be found in Table 3.

**Table 1:** The sensor streams in the released dataset, and the units which they are measured in. Uncertainty is reported as specified in the technical product sheets.

Sensor	Description	Unit	Uncertainty
CO_2_	CO_2_ concentration by wall mounted sensor.	Parts Per Million (ppm)	300–1000 ppm: ±120 ppm
			1000–2000 ppm: ±250 ppm
			2000–5000 ppm: ±300 ppm
Relative Humidity	Relative humidity measured by wall mounted sensor.	Percentage	±5%
Illuminance	Illuminance measured by ceiling mounted sensor.	Lux	Operating range 10–2000 lux
Occupant count	Estimated number of occupants in the room.	Count	Not available
Enter door 1	Number of counted occupants entering since last reading.	Count	1%
Exit door 1	Number of counted occupants leaving since last reading.	Count	1%
Enter door 2	Number of counted occupants entering since last reading.	Count	1%
Exit door 2	Number of counted occupants leaving since last reading.	Count	1%
Temperature	Temperature in the room measured by wall mounted sensor.	Degree Celsius	1 °C
VAV damper position	The openness of the variable air volume damper for in-flow air ventilation.	Percentage	Not available

**Table 2:** The sampling strategy for each of the sensor streams at the individual rooms. CO_2_ is sampled using dynamic sampling rate (DSR), where the sampling frequency is increased (up to one sample per minute) when higher levels of CO_2_ are observed. Occupant counts are sampled using a static sampling rate (SSR). The relative humidity and temperature streams are sampled using a threshold-based strategy. The variable air volume (VAV) damper position is sampled when there is a change in the position.

Sensor	Room 1	Room 2	Room 3
CO_2_	DSR (5 minutes interval in normal operation).	DSR (5 minutes interval in normal operation).	DSR (5 minutes interval in normal operation).
Relative humidity	Threshold based (≈2% change).	Threshold based (≈2% change).	Threshold based (≈2% change).
Illuminance	SSR (1 minutes interval).	SSR (1 minutes interval).	SSR (1 minutes interval).
Occupant counts	SSR (1 minutes interval).	SSR (1 minutes interval).	SSR (1 minutes interval).
Enter door 1	SSR (1 minutes interval).	SSR (1 minutes interval).	SSR (1 minutes interval).
Exit door 1	SSR (1 minutes interval).	SSR (1 minutes interval).	SSR (1 minutes interval).
Enter door 2	SSR (1 minutes interval).	SSR (1 minutes interval).	SSR (1 minutes interval).
Exit door 2	SSR (1 minutes interval).	SSR (1 minutes interval).	SSR (1 minutes interval).
Temperature	Threshold based (≈0.1° change).	Threshold based (≈0.1° change).	Threshold based (≈0.1° change).
VAV damper position	Sample on change.	Sample on change.	Sample on change.

**Table 3:** Overview of the locations of the sensors inside the room.

Sensor	Room 1	Room 2	Room 3
CO_2_	Installed on south facing wall, in 180 cm.	Installed on south facing wall, in 180 cm.	Installed on south facing wall, in 180 cm.
Illuminance	Installed on the ceiling.	Installed on the ceiling.	Installed on the ceiling.
Occupant counts	Virtual sensor.	Virtual sensor.	Virtual sensor.
Enter door 1	Installed on the ceiling. In the hallway 0.5 m from door 1.	Installed on the ceiling. In the hallway 0.5 m from door 1.	Installed on the ceiling. In the hallway 0.5 m from door 1.
Exit door 1	Installed on the ceiling. In the hallway 0.5 m from door 1.	Installed on the ceiling. In the hallway 0.5 m from door 1.	Installed on the ceiling. In the hallway 0.5 m from door 1.
Enter door 2	Installed on the ceiling. In the hallway 0.5 m from door 2.	Installed on the ceiling. In the hallway 0.5 m from door 2.	Installed on the ceiling. In the hallway 0.5 m from door 2.
Exit door 2	Installed on the ceiling. In the hallway 0.5 m from door 2.	Installed on the ceiling. In the hallway 0.5 m from door 2.	Installed on the ceiling. In the hallway 0.5 m from door 2.
Temperature	Installed on the south-facing wall, at height 180 cm.	Installed on the south-facing wall, at height 180 cm.	Installed on the south-facing wall, at height 180 cm.
VAV damper position	On the damper.	On the damper.	On the damper.

## Methods

### Selection methodology

The published dataset is collected in the period of March 1st, 2018, to April 30th, 2019. We would like only to publish periods of continuous readings, but since the source is a real building and BMS APIs were used for collecting the data, we have had to adjust expectations slightly. We only considered full days of data. Days where the CO_2_ sampled stream had more than three missing readings in a row were not considered, hence allows gaps of 15 minutes. Threshold-based sensors which only collect a sample when there is a change larger than the threshold has not been conceded for eliminating days since it is impossible to evaluate how many readings such streams should contain. Additionally, we chose not to consider two consecutive days as this would make the released data susceptible to privacy attacks. This decision is based on the results of a study^11^, which showed that using CO_2_ streams, the data could be deanonymized. This attack could be used to identify the weekday. Which could be used to reveal the identity of the room, by doing a data linkage attack using the teaching rooms scheduled activates and the released streams, as showcased in^11^. To eliminated days in a sequence, we have selected the following procedure: For sequences with an even amount of days, the days were randomly removed to comply with the rule. Uneven amount of days in the sequence was removed by maximizing the number of days in the output. This left 44 full days of data covering the three test rooms and all the sensor modalities. These make up the days of the released dataset.

### Data processing

In the released dataset, we have provided two forms of the data, the original raw form and one which has been pre-processed to allow easier use of the data by having a stable sample rate. The pre-processing applies forward fill and then backward fill on the original streams. Forward fill, fills missing samples for the desired sampling frequency by filling the gaps in the stream with the last reported value in the stream, until a new reported value is reacted in the stream, backward fills do the same but back in time. The sampling rate for the fills has been set to minute-wise sampling for all of the streams. Other sampling rates can be computed using the original dataset. The sample rate has been selected to accommodate the identified use cases, e.g., occupancy and model-predictive control^9^. The original dataset has only been changed for the event and threshold-based sensors, by adding a reading at 00:00:00, which had the value of the last reading of the previous day.

### Data suppression

The most sensitive part of the data release is the identity of the rooms. Since combined with the occupant counts and knowledge of room activities, one can calculate a teacher performance index, as demonstrated in^11^. Thus we have anonymized room identity and replaced the dates by a DayId, which is a random number assigned in a non-chronological order. To limit the effect on the usefulness of the dataset, we have introduced a year, month, and a workday indicator. The time of day is untouched.

## Data Records

**Fig. 1:**
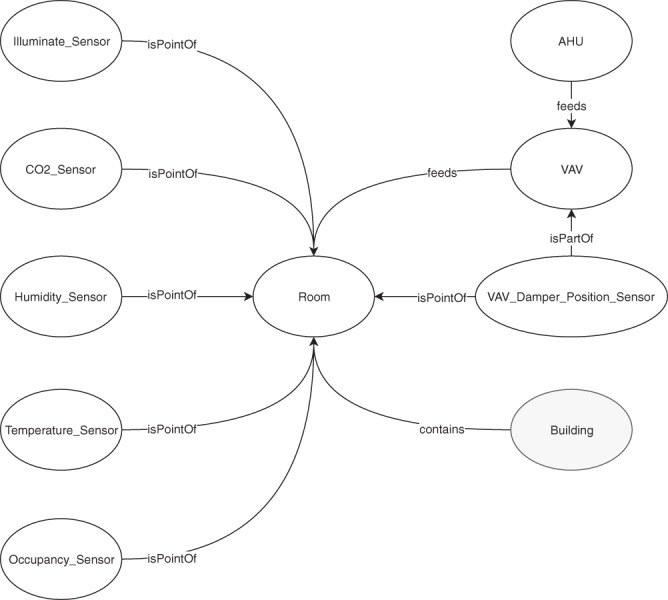
Visual representation of the relationships defined on each room. All rooms are attached to the same building, but no two rooms are served by the same AHU nor VAV damper position. Note the omission of floors between the rooms and the building.

The released dataset, hosted on figshare^12^, contains the mentioned sensor modalities, and the amount of readings of each of the rooms can be seen in Table 4. The upsampled dataset 67680 readings per stream. The data coverage for the sensors using the sampling strategies of dynamic sampling rate (DSR) and static sampling rate (SSR), can be found in Table 5. The illuminance data stream have relatively low data coverage, we have added it since it still captures the tendency for the light level in the rooms through the days, although the coverage can affect the usefulness of the stream. Summary statistics for the upsampled dataset for rooms 1, 2 and, 3 can be found in Tables 6–8. The data for each combination of sensor modality and room can be found in separate comma-separated value (CSV) files. In addition to the sensor values, these files have columns for the metadata defined in the data suppression section, namely: Timestamps, year, month and workday indicators, and dayId, which also can be found in the readme file. The two versions of the data, original and upsampled, can be found in the folder’s original and filleddata, respectively. The metadata also contains room type, size, seating capacity, and volume, which can be found in Table 9, and in the roominfo file. Furthermore, we have provided a Brick representation of the sensor instrumentation^13^, found in the brick_graph file generated using the brick_generator script. The Brick model consists of the physical relations between the sensors streams, the building, and rooms. It is modeled using Resource Description Framework (RDF) triples between the elements in the model. Each of the rooms models are the same, an example of them can be found in Fig. 1. Finally, have we included a categorization of the sensors following the Mahdavi and Taheri^14^ ontology, which can be found in the occupant-behavior-ontology file or in Tables 10, 11, and 12 for the indoor conditions, inhabitants, and control systems, respectively.

**Table 4:** The sensor modalities in each of the rooms and the number of readings in the streams, in the original data stream.

Sensor	Room 1 number of readings	Room 2 number of readings	Room 3 number of readings
CO_2_	12044	11897	12059
Relative humidity	6440	7639	5468
Illuminance	24813	23663	21542
Occupant counts	63350	63350	63350
Enter door 1	63334	63334	63288
Exit door 1	63334	63334	63288
Enter door 2	63170	63287	63335
Exit door 2	63170	63287	63335
Temperature	5200	2457	2352
VAV damper position	880	659	656

**Table 5:** The data coverage, for the sensors with sampling strategy of DSR and SSR, in the original data stream.

Sensor	Room 1 data coverage	Room 2 data coverage	Room 3 data coverage
CO_2_	93.19%	92.42%	93.77%
Illuminance	39.16%	37.35%	34.00%
Occupant counts	99.98%	99.98%	99.98%
Enter door 1	99.96%	99.96%	99.88%
Exit door 1	99.96%	99.96%	99.88%
Enter door 2	99.70%	99.89%	99.96%
Exit door 2	99.70%	99.89%	99.96%

**Table 6:** Summary statistics for the upsampled streams in Room 1.

Sensor	Min value	Max value	Standard deviation	Skew	Kurtosis
CO_2_	406.72	1046.40	113.49	1.46	1.57
Relative humidity	22.75	74.90	9.90	0.45	−0.35
Illuminance	0	2064.64	237.52	2.50	10.90
Occupant counts	0	80	15.87	2.14	3.71
Enter door 1	0	36	0.56	15.01	605.39
Exit door 1	0	23	0.59	12.42	256.30
Enter door 2	0	41	0.88	8.14	156.94
Exit door 2	0	43	0.97	9.46	171.51
Temperature	19.40	28.40	1.50	0.48	0.21
VAV damper position	0%	100%	29.20	1.10	0.10

**Table 7:** Summary statistics for the upsampled streams in Room 2.

Sensor	Min value	Max value	Standard deviation	Skew	Kurtosis
CO_2_	405.76	1160.96	81.72	2.01	5.86
Relative humidity	22.75	75.68	10.10	0.56	−0.04
Illuminance	0	2005.76	165.12	4.10	30.29
Occupant counts	0	65	6.47	2.38	7.54
Enter door 1	0	8	0.29	9.92	135.57
Exit door 1	0	14	0.33	10.76	176.40
Enter door 2	0	12	0.43	8.79	108.80
Exit door 2	0	12	0.42	8.95	113.54
Temperature	17.30	26.50	1.18	0.71	1.34
VAV damper position	0%	100%	17.79	1.51	1.41

**Table 8:** Summary statistics for the upsampled streams in Room 3.

Sensor	Min value	Max value	Standard deviation	Skew	Kurtosis
CO_2_	408.0	1207.68	102.34	1.76	4.95
Relative humidity	22.75	70.98	10.16	0.36	−0.47
Illuminance	0	1561.60	117.97	2.53	16.83
Occupant counts	0	80	6.83	3.20	15.82
Enter door 1	0	19	0.47	11.26	211.68
Exit door 1	0	14	0.44	9.33	126.36
Enter door 2	0	6	0.23	10.76	155.78
Exit door 2	0	9	0.27	11.22	174.45
Temperature	15.70	24.70	0.92	0.87	1.76
VAV damper position	0%	100%	28.26	1.22	0.39

**Table 9:** Metadata for each of the rooms.

Room Name	Room type	Size of the room	Seating capacity	Volume
Room 1	lecture room	139 m^2^	84	461.48 m^3^
Room 2	study zone	125 m^2^	32	418.75 m^3^
Room 3	study zone	125 m^2^	32	418.75 m^3^

**Table 10:** Sensor streams monitoring indoor conditions. For the upsampled version of the dataset. Short names used in the table: Spatial attribute (SA), Temporal attribute (TA), Topological reference (TR), Sampling Interval (SI), Data Source (DS), and Quantitative (Q).

Categories of measured data	Subcateg-ories of measured data	Specific variables measured	Measured value	Data type	Unit	SA/TR	TA/SI	DS/Categ-ory
Indoor conditions	Indoor Air Quality	Indoor Air Quality − CO_2_ concentration	1	Q	ppm	Room 1	1 min	Sensor
Indoor conditions	Indoor Air Quality	Indoor Air Quality − CO_2_ concentration	1	Q	ppm	Room 2	1 min	Sensor
Indoor conditions	Indoor Air Quality	Indoor Air Quality − CO_2_ concentration	1	Q	ppm	Room 3	1 min	Sensor
Indoor conditions	Hygro-thermal	Indoor Air Quality − Air Temperature	1	Q	celsius	Room 1	1 min	Sensor
Indoor conditions	Hygro-thermal	Indoor Air Quality − Air Temperature	1	Q	celsius	Room 2	1 min	Sensor
Indoor conditions	Hygro-thermal	Indoor Air Quality − Air Temperature	1	Q	celsius	Room 3	1 min	Sensor
Indoor conditions	Hygro-thermal	Indoor Air Quality − CO_2_ concentration	1	Q	%	Room 1	1 min	Sensor
Indoor conditions	Hygro-thermal	Indoor Air Quality − CO_2_ concentration	1	Q	%	Room 2	1 min	Sensor
Indoor conditions	Hygro-thermal	Indoor Air Quality − CO_2_ concentration	1	Q	%	Room 3	1 min	Sensor
Indoor conditions	Hygro-thermal	Indoor Air Quality − CO_2_ concentration	1	Q	lux	Room 1	1 min	Sensor
Indoor conditions	Visual	Indoor Air Quality − CO_2_ concentration	1	Q	lux	Room 2	1 min	Sensor
Indoor conditions	Visual	Indoor Air Quality − CO_2_ concentration	1	Q	lux	Room 3	1 min	Sensor

**Table 11:** Sensor streams monitoring inhabitants. For the upsampled version of the dataset. Short names used in the table: Spatial attribute (SA), Temporal attribute (TA), Topological reference (TR), Sampling Interval (SI), Data Source (DS), and Quantitative (Q).

Categories of measured data	Subcateg-ories of measured data	Specific variables measured	Measured value	Data type	Unit	SA/TR	TA/SI	DS/Categ-ory
Inhabitants	Position	Position − 3D Camera-based occupancy	1	Q	persons	Room 1	1 min	Sensor
Inhabitants	Position	Position – 3D Camera-based occupancy	1	Q	persons	Room 2	1 min	Sensor
Inhabitants	Position	Position − 3D Camera-based occupancy	1	Q	persons	Room 3	1 min	Sensor
Inhabitants	Position	Position − Enter door 1	1	Q	persons	Room 1	1 min	Sensor
Inhabitants	Position	Position − Enter door 1	1	Q	persons	Room 2	1 min	Sensor
Inhabitants	Position	Position − Enter door 1	1	Q	persons	Room 3	1 min	Sensor
Inhabitants	Position	Position − Enter door 1	1	Q	persons	Room 1	1 min	Sensor
Inhabitants	Position	Position − Enter door 1	1	Q	persons	Room 2	1 min	Sensor
Inhabitants	Position	Position − Enter door 1	1	Q	persons	Room 3	1 min	Sensor
Inhabitants	Position	Position − Enter door 2	1	Q	persons	Room 1	1 min	Sensor
Inhabitants	Position	Position − Enter door 2	1	Q	persons	Room 2	1 min	Sensor
Inhabitants	Position	Position − Enter door 2	1	Q	persons	Room 3	1 min	Sensor
Inhabitants	Position	Position − Enter door 2	1	Q	persons	Room 1	1 min	Sensor
Inhabitants	Position	Position − Enter door 2	1	Q	persons	Room 2	1 min	Sensor
Inhabitants	Position	Position − Enter door 2	1	Q	persons	Room 3	1 min	Sensor

**Table 12:** Sensor streams monitoring control systems. For the upsampled version of the dataset. Short names used in the table: Spatial attribute (SA), Temporal attribute (TA), Topological reference (TR), Sampling Interval (SI), Data Source (DS), and Quantitative (Q).

Categories of measured data	Subcateg-ories of measured data	Specific variables measured	Measured value	Data type	Unit	SA/TR	TA/SI	DS/Categ-ory
Control systems/devices	Ventilation	Ventilation–Damper position (per room)	1	Q	%	Room 1	1 min	Sensor
Control systems/devices	Ventilation	Ventilation –Damper position (per room)	1	Q	%	Room 2	1 min	Sensor
Control systems/devices	Ventilation	Ventilation –Damper position (per room)	1	Q	%	Room 3	1 min	Sensor

## Technical Reliability

To evaluate the technical reliability of the dataset, we have provided plots, showing the daily profiles of each sensor modality. Furthermore, we provide additional evidence for each of the streams based on statistical analysis.

**Fig. 2:**
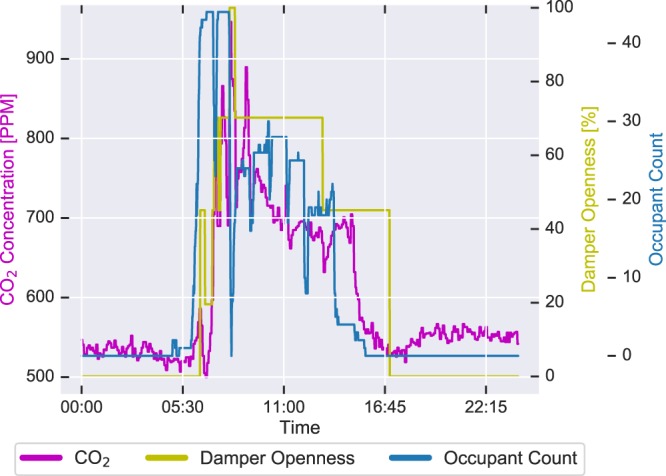
The relationship between the number of occupants, the VAV damper openness of the heating ventilation and air conditioning (HVAC) system, and the CO_2_ conditions, in room 1 on the day with the DayId of 9.

In^15^, the authors showcase the relation between the VAV damper position, CO_2_, and the number of occupants, which can be seen in Fig. 2. The Pearson product-moment correlation coefficients, which measure the linear association between two variables, between the CO_2_ and damper position in the dataset is 0.87700, 0.89287, and 0.80668 for Room 1, 2 and 3, respectively. The correlation between CO_2_ and the number of occupants is 0.65863, 0.83680, and 0.81663 for the rooms. Finally, the correlation coefficient between damper position and the number of occupants is 0.70158, 0.77950, and 0.69057 in the dataset, using the data form the day with the DayId of 9. These numbers highlight the expected relationships between these modalities^16^. As expected, there exists a slight positive correlation among these data streams. This is because the operations of the damper position are regulated to maintain CO_2_ concentration below a particular threshold. Likewise, CO_2_ concentration is mostly influenced by the number of occupants in a particular space. However, we do not expect correlation coefficients for a perfect relationship. We have compared the total amount of people entering and exiting the three rooms. The results show that, according to the sensors, 0.17% more enters room 1 then leaves the room. In room 2, 1.12%  more leave the room than enters the room. In room 3, 0.29% more leave the room than enters. For all rooms, we have used the total amount of people entering and exiting in the monitored period for the comparison. These numbers indicate that the observed sensing error is very low.

**Fig. 3:**
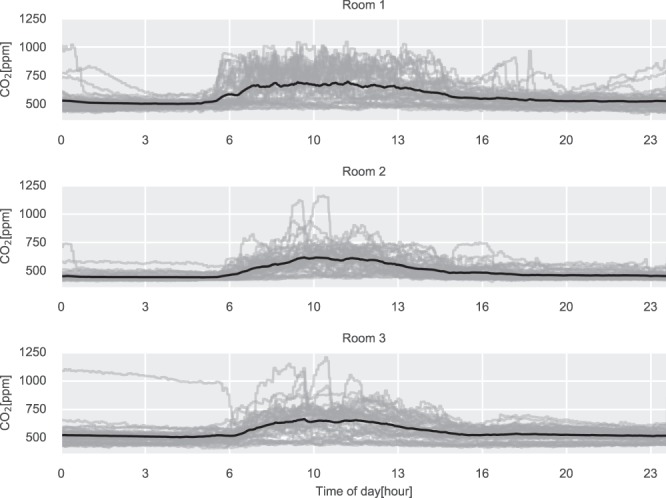
Daily CO_2_ concentrations profiles for the three test rooms. The gray lines are daily profiles, the black line is the average daily profile.

**Fig. 4:**
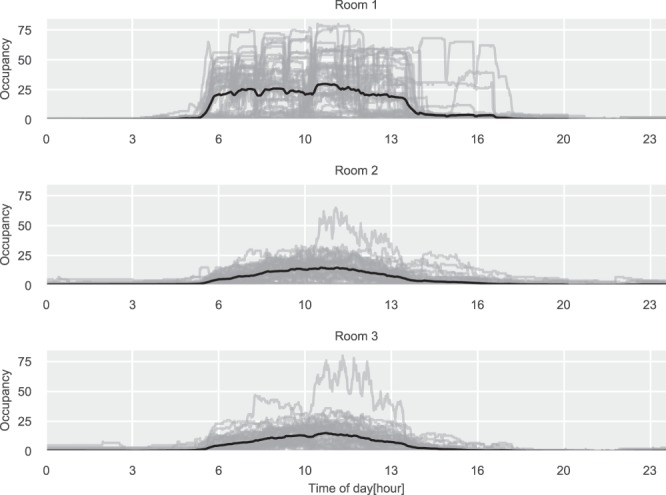
Daily occupancy estimations for the three test rooms. The gray lines are daily profiles, the black line is the average daily profile.

**Fig. 5:**
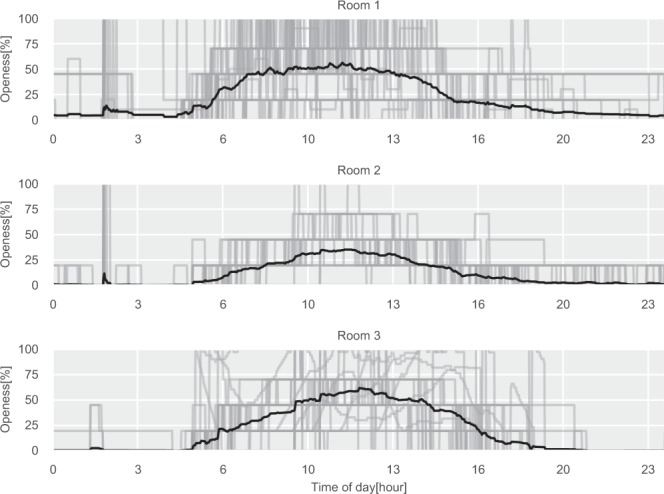
Daily damper poisons profiles for the three test rooms. The gray lines are daily profiles, the black line is the average daily profile.

In^17^ there have been performed reliability tests for the CO_2_ and temperature streams, where it was found that the CO_2_ sensors were not calibrated and therefore was the sensors replaced and calibrated. In Figs. 3–5 the profiles for the CO_2_ concentrations, the damper position, and the occupancy estimation for the rooms can be seen. Highlighting expected patterns during daytime versus night. The lowest CO_2_ readings for the three rooms are 406.72, 405.76, and 408.0 ppm for Room 1, 2, and 3, respectively. This is close to the ambient concentrations for Denmark, which is around 400 ppm.

**Fig. 6:**
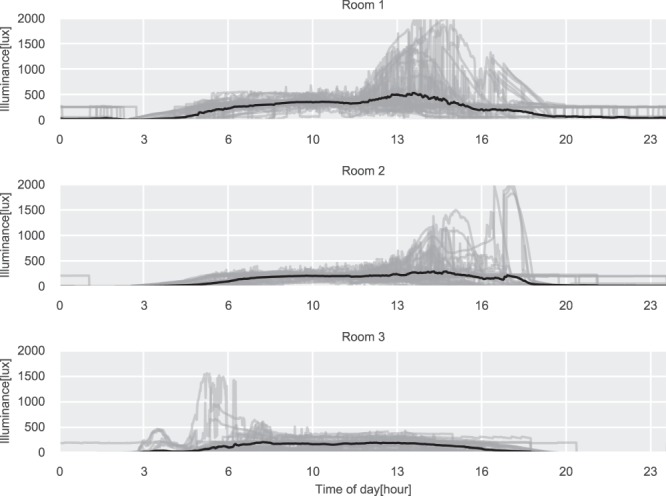
Daily illuminance profiles for the three test rooms. The gray lines are daily profiles, the black line is the average daily profile.

**Fig. 7:**
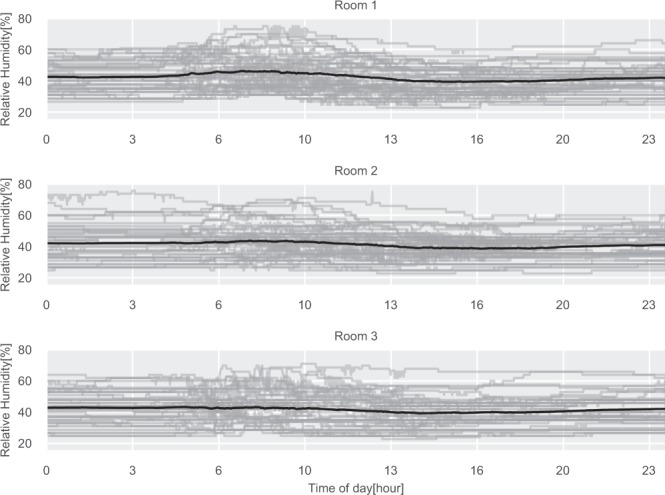
Daily relative humidity profiles for the three test rooms. The gray lines are daily profiles, the black line is the average daily profile.

**Fig. 8:**
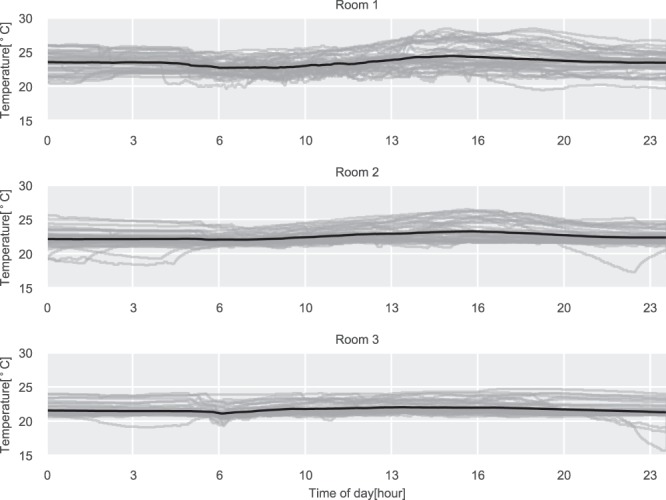
Daily temperature profiles for the three test rooms. The gray lines are daily profiles, the black line is the average daily profile.

The daily illuminance level for the three rooms is shown in Fig. 6. Rooms 1 and 2 are both located on the west side of the building, which can be inferred by observing the daily profiles shown in the figure, where the illuminance is peaking later during the day when there is direct sunlight on the windows. The same can be observed for Room 3, which has eastern exposure and therefore peaks in the morning. Furthermore, does all three rooms have the lowest reading of 0 lux, which is the expected lowest value since the sensor can not detect values below 10 lux, as specified in the technical product sheet. The humidity and temperature daily profiles can be seen in Figs. 7 and 8. Here the impact of the sun in the afternoon for Room 1 and Room 2 can be observed, which is not present for Room 3.

## Data Availability

The pre-processing code for suppression and data processing is available online (https://github.com/sdu-cfei/Building-Data-Occupant-Modeling).
