# Publisher Correction: Room-level occupant counts and environmental quality from heterogeneous sensing modalities in a smart building

**DOI:** 10.1038/s41597-020-0416-8

**Published:** 2020-02-28

**Authors:** Jens Hjort Schwee, Aslak Johansen, Bo Nørregaard Jørgensen, Mikkel Baun Kjærgaard, Claudio Giovanni Mattera, Fisayo Caleb Sangogboye, Christian Veje

**Affiliations:** University of Southern Denmark, Campusvej 55, 5230 Odense, Denmark

Correction to: *Scientific Data* 10.1038/s41597-019-0274-4, published online 26 November 2019

Following publication, it was found that a number of errors were introduced into Table 10 of this Data Descriptor during the typesetting process. These errors have now been corrected in both the HTML and PDF versions.

